# Mechanisms and Functional Significance of Stroke-Induced Neurogenesis

**DOI:** 10.3389/fnins.2015.00458

**Published:** 2015-12-08

**Authors:** Quentin Marlier, Sebastien Verteneuil, Renaud Vandenbosch, Brigitte Malgrange

**Affiliations:** GIGA-Neurosciences, University of Liege, C.H.U. Sart TilmanLiege, Belgium

**Keywords:** stroke, neurogenesis, niches, stem cells, therapy

## Abstract

Stroke affects one in every six people worldwide, and is the leading cause of adult disability. After stroke, some limited spontaneous recovery occurs, the mechanisms of which remain largely unknown. Multiple, parallel approaches are being investigated to develop neuroprotective, reparative and regenerative strategies for the treatment of stroke. For years, clinical studies have tried to use exogenous cell therapy as a means of brain repair, with varying success. Since the rediscovery of adult neurogenesis and the identification of adult neural stem cells in the late nineties, one promising field of investigation is focused upon triggering and stimulating this self-repair system to replace the neurons lost following brain injury. For instance, it is has been demonstrated that the adult brain has the capacity to produce large numbers of new neurons in response to stroke. The purpose of this review is to provide an updated overview of stroke-induced adult neurogenesis, from a cellular and molecular perspective, to its impact on brain repair and functional recovery.

## Introduction

Stroke is the second leading cause of death, the most common cause of adult-acquired disability and affects one in every six people worldwide (Moskowitz et al., 2010). The number of people who survive a stroke is increasing, and with an aging population, the incidence and prevalence of stroke are predicted to rise even more (Sun et al., 2012). Despite years of research, effective treatments remain elusive. Currently, the only proven therapy for acute ischemic stroke is systemic thrombolysis with recombinant tissue plasminogen activator (rtPA). To be effective, rtPA must be administered within a maximum of 4.5 h after the symptoms first start. This short timeframe and potential adverse effects have limited the use of rtPA to 3–5% of stroke patients (Ruan et al., 2015). Grafting stem cells represents a compelling alternative and offers both a wide array and an unlimited supply of cells. Indeed, the transplantation of neural stem cells (NSCs), mesenchymal stem cells (MSCs), embryonic stem cells (ESCs), or induced pluripotent stem cells (iPSCs) could be used to replace neuronal loss after stroke (Kalladka and Muir, 2014). However, exogenous stem cell therapy has both technical and ethical issues. For instance, cell survival and migration rely heavily on the timing and mode of delivery (Li et al., 2010; Darsalia et al., 2011). Moreover, surgical procedure and toxicity (as cancer induction) increase the complexity of transplanted cell therapies (Kawai et al., 2010; Ben-David and Benvenisty, 2011). Finally, some ethical issues may arise from the use of fetal/embryonic cells.

Despite the fact that the central nervous system (CNS) has a limited repair capacity (Nakagomi et al., 2011), some degree of spontaneous recovery from brain ischemia invariably occurs (Yu et al., 2014). This repair process involves neurogenesis, angiogenesis, and axonal sprouting and synaptogenesis. Here we concentrate on the events that are associated with the production of new neurons and not the mechanisms that involve the reorganization of connectivity among surviving neurons, which is reviewed elsewhere (Jones and Adkins, 2015).

Recent experimental findings have raised the possibility that functional improvement after stroke may be induced through neuronal replacement by endogenous NSCs. Indeed, the original dogma that no new neurons are formed after birth has been definitively overturned during the past few decades. The discovery of the thymidine analog bromodeoxyuridine (BrdU)—that incorporates into DNA in S-phase and can be detected by immunohistochemistry—has allowed researchers to conclusively demonstrate the generation of new neurons in the brain of all adult mammals including humans (Eriksson et al., 1998; Gage, 2000). This production of new neurons in the adult brain—so-called adult neurogenesis—takes place in areas called neurogenic niches. The subventricular zone (SVZ) of the lateral ventricle and the subgranular zone (SGZ) of the dentate gyrus (DG) are the two main neurogenic niches containing adult NSCs that proliferate, divide and differentiate into mature neurons. Recently, new evidence have highlighted that adult neurogenesis could also takes place in other brain areas, along the ventricular system, mostly in pathological conditions (Lin and Iacovitti, 2015).

The capacity to produce new neurons in the adult brain and the ability of the ischemia-injured adult brain to partially recover suggest a possible relationship between adult neurogenesis and stroke recovery. Indeed, many studies have shown an increase in cell proliferation in the rodent SVZ following ischemic injury (Thored et al., 2006), and evidence for stroke-induced neurogenesis in the human brain has also been reported (Jin et al., 2006). In addition, endogenous brain repair is not limited to neurogenic niches. Recent studies have shown that glial cells surrounding the infarct core can be reactivated following ischemia. Indeed, pericytes, oligodendrocyte precursors, and astrocytes are all able to differentiate into neurons following brain injury (Robel et al., 2011; Heinrich et al., 2014; Nakagomi et al., 2015; Torper et al., 2015). Moreover, surviving neurons may reorganize their connections in a manner that supports some degree of spontaneous improvement. Therefore, a promising field of investigation is focused on triggering and stimulating this self-repair system to replace dead neurons following an ischemic attack.

## Stroke pathophysiology

Stroke, also known as cerebrovascular accident, results from a transient or permanent reduction in cerebral blood flow that is restricted to the territory of a major brain artery (Woodruff et al., 2011). The two main types of stroke are hemorrhagic stroke (15%) due to bleeding and ischemic stroke (85%) due to lack of blood flow. The three main mechanisms causing ischemic stroke are thrombosis, embolism and global ischemia (Hossmann, 2012). In all of these cases, decreased or absent circulating blood deprives neurons of their necessary substrates, leading irrevocably to cell death. Although different brain regions have varying thresholds for ischemic cell damage, and certain populations of neurons are selectively more resistant to ischemia, neurons are by far the most sensitive cells, ahead of oligodendrocytes, astrocytes, and vascular cells (Woodruff et al., 2011). Despite the fact that neurological dysfunction occurs within seconds to minutes after decreased perfusion, the evolution of ischemic injury continues for several hours and even days (Moskowitz et al., 2010), leading to massive neuronal death and corresponding patient disabilities.

Acute occlusion of a major brain artery leads to spatially and temporally stereotyped events, including morphological damage that evolves over a prolonged period and which depends on the topography, severity and duration of ischemia (Hossmann, 2012). Two specific areas of damage can be defined. The first area is the rapidly formed “ischemic core,” which corresponds to the irreversibly damaged tissue close to the occluded artery (Yuan, 2009). It is characterized by a < 20% of baseline blood flow levels, below the energy metabolism threshold (Lo, 2008). In this area, neurons are deprived of their two main supplies: glucose and oxygen. Without these two factors, neurons are unable to produce the ATP needed to maintain ionic gradients. This energy deficit also induces the activation of voltage-gated calcium (Ca^2+^) channels. As a consequence, an increase in intracellular Ca^2+^ concentration is observed, leading to the activation of phospholipases and proteases, which degrade membranes and proteins essential for cell integrity. Moreover, this massive accumulation of Ca^2+^ is passively followed by water, causing intracellular edema and cell swelling (Iadecola and Anrather, 2011). Unless blood flow is quickly restored, necrotic and irreversible cell death will consequently occur within minutes or a few hours. The second area is the “ischemic penumbra,” a region of moderately perfused tissue surrounding the ischemic core, where oxygen is still present to irrigate neurons thanks to collateral arteries. In this area, the reduction of blood flow is not sufficient to cause energy failure and disrupt ionic gradients. Consequently, neurons remain viable but are stressed and vulnerable. Excessive extracellular accumulation of glutamate is a major factor contributing to cell death in the ischemic penumbra zone. The resulting overactivation of glutamate receptor channels of the N-methyl-D-aspartate (NMDA) subtype leads to cytoplasmic accumulation of Ca^2+^, which activates Ca^2+^-dependent enzymes, including calpains and caspases, and finally leads to apoptotic cell death (Iadecola and Anrather, 2011). Moreover, during ischemia, mitochondria generate reactive oxygen species (ROS), which modulate signal transduction cascades that tip the balance between pro-death and pro-survival pathways, or act directly as executioners of cell death (Moskowitz et al., 2010). The importance of cell death in the ischemic penumbra area depends upon residual cerebral blood flow (CBF). If CBF is not re-established rapidly, the neurons will die after a few hours or days. The ischemic penumbra represents a salvageable brain area, in which neuronal activity is suppressed but the tissue is potentially viable (Moskowitz et al., 2010). Therefore, early reperfusion is the major target of most therapeutic and experimental interventions in an attempt to reestablish sufficient CBF and rescue cells in the penumbra.

In addition to the above-mentioned histological features, focal ischemia leads to a robust inflammatory response that begins within a few hours. Early on, the production of cytokines by vascular cells and perivascular microglia exposed to ischemic insult contribute to the inflammatory response. Indeed, activated microglia produce toxic metabolites such as ROS and nitric oxide (NO), as well as pro-inflammatory mediators such as IL-6, matrix metalloproteinases (MMPs) and toll-like receptors, which contribute to extend brain injury (Schilling et al., 2003; del Zoppo et al., 2007; Okun et al., 2009). Although activated microglia and vascular cells appear detrimental to cell survival and recovery at the beginning of the insult, chronically activated microglia may, however, secrete beneficial factors that enhance tissue repair (Moskowitz et al., 2010). Reactive astrocytes also have a biphasic role regarding the inflammatory response to stroke. They can extend ischemic lesions by producing pro-inflammatory cytokines (del Zoppo et al., 2000), but also possess a neuroprotective role, through the release of erythropoietin, which leads to the phosphorylation and subsequent inactivation of the pro-apoptotic protein BAD (Ruscher et al., 2002; Prass et al., 2003). This could be of importance regarding spontaneous recovery and regulation of stroke-induced neurogenesis, as discussed below.

## Therapeutic strategies for stroke

The development of therapeutic strategies aimed at overcoming neuronal loss, especially by avoiding delayed neuronal death in penumbra or by replacing dead cells in the ischemic core, is essential. Despite significant improvements after systemic thrombolysis using rtPA, only a small number of patients have timely access to this therapy. As an alternative, the most encouraging approach is stem cell therapy, by using exogenous stem cell grafts or stimulating endogenous stem cell proliferation and differentiation into cells of interest.

### Exogenous stem cells

Cell replacement for stroke requires the regeneration of multiple functionally specialized cell types, including different kind of neurons, glial and endothelial cells, to restore the entire neurovascular unit (Kalladka and Muir, 2014). Grafting cells into the CNS represents a promising avenue for cell replacement therapies. Grafted cells could potentially differentiate into specific cell subtypes and functionally integrate into the host circuitry in order to repopulate damaged areas. Different sources of stem cells have been proposed to treat stroke and include NSCs, ESCs, iPSCs, and MSCs (Table 1).

**Table 1:** **Summary of exogenous stem cells types and their role in stroke recovery**.

	**NSC**	**ESC**	**iPSC**	**MSC**
Origin	ESC iPSC Skin or blood adult stem cells	• Blastocyst inner cell mass	• Autologous patient-specific cells	• Bone marrow stromal cells
Mode of administration	ST IV IA ICV	• ST	• ST	IV ST
Behavior	Proliferate, migrate and differentiate into neurons Stimulate endogenous stem cell proliferation Modulate inflammation response	• Differentiate into neurons	• Differentiate into neurons	Migrate into ischemic area Modulate inflammatory response
Therapeutic outcome	• Promote functional recovery	• Promote functional recovery	• Little functional impact	• Promote functional recovery
Advantages	Standardized isolation Good survival	• Culture expansion	• No ethical, moral and legal issues	Facility to acquire No ethical issue
Limitations	Dependence of timing and mode of delivery Reproducibility	Ethical issues Toxicity Reproducibility	Toxicity Reproducibility	• Poor differentiation • Reproducibility
References	Anderson, 2001; Chu et al., 2005; Jiang et al., 2006; Martino and Pluchino, 2006; Bacigaluppi et al., 2009; Zhang and Chopp, 2009; Barkho and Zhao, 2011; Darsalia et al., 2011; Martino et al., 2011; Song et al., 2011; Hassani et al., 2012; Kokaia et al., 2012; Oki et al., 2012; Jensen et al., 2013; Mine et al., 2013; Hermann et al., 2014; Tang et al., 2014	Bühnemann et al., 2006; Daadi et al., 2008; Ben-David and Benvenisty, 2011	Kawai et al., 2010; Ben-David and Benvenisty, 2011; Jiang et al., 2011; Oki et al., 2012; Jensen et al., 2013	Li et al., 2000; Chen et al., 2001a,b; Chen et al., 2008; Kang et al., 2003; Kurozumi et al., 2005; Honma et al., 2006; Horita et al., 2006; Cui et al., 2007; Andrews et al., 2008; Koh et al., 2008; Bao et al., 2011, 2013; Gutiérrez-Fernández et al., 2011; Braun et al., 2012; Ruan et al., 2013; Wang et al., 2014; Lee et al., 2015

*ESC, embryonic stem cells; IA, intra-arterial; ICV, intracerebroventricular; iPSC, induce-pluripotent stem cells; IV, intravenous; MSC, mesenchymal stem cells; NSC, neural stem cells; ST, stereotaxic*.

NSCs are self-renewing, multipotent cells with the ability to proliferate and give rise to neurons, astrocytes and oligodendrocytes, *in vitro* and *in vivo*. They can be derived from ESCs or iPSCs, but also directly from adult stem cells located and isolated from different tissues, such as skin or blood (Anderson, 2001; Morrison, 2001). Recently, transplantation of NSCs from the adult murine brain in ischemic rats led to cell migration to ischemic regions and significant behavioral improvements compared to non-transplanted animals, although many cells died early after transplantation (Chu et al., 2005; Jiang et al., 2006; Zhang and Chopp, 2009; Darsalia et al., 2011; Song et al., 2011). Cell survival and behavior is strongly influenced by the timing and mode of their delivery. For example, intraparenchymal transplantation decreases sensorimotor dysfunctions and motor deficits, while intracerebroventricular injection does not result in any improvement (Smith et al., 2012). Moreover, upon intravenous transplantation of adult mouse NSCs after stroke, only a few percent of cells survive in the brain (Bacigaluppi et al., 2009). Importantly, exogenous stem cell therapy could enhance the endogenous self-repair system. Indeed, transplanted human NSCs, by releasing several factors such as vascular endothelial growth factor (VEGF), neurotrophins or fibroblast growth factor-2 (FGF-2), are highly effective in stimulating endogenous neurogenesis in rats when cells are delivered directly into the ischemic brain parenchyma (Sun et al., 2003; Türeyen et al., 2005; Drago et al., 2013). Finally, this improved endogenous neurogenesis is accompanied by modulation of the inflammatory response. Transplantation of adult NSCs induces a down-regulation of pro-inflammatory mediators, such as interferon-γ (IFN-γ), tumor necrosis factor alpha (TNF-α) and interleukin-1β (IL-1β) in ischemic mice brains (Bacigaluppi et al., 2009). This drastically decreases the microglia driven inflammatory response (Oki et al., 2012) and therefore host-driven repair (Martino and Pluchino, 2006; Martino et al., 2011; Kokaia et al., 2012).

ESCs are pluripotent cells derived from the inner cell mass of blastocysts. The major advantage of ESCs is that a large number of cells can be expanded in culture and differentiated in any neuronal subtype. However, major ethical (embryos destruction) and scientific (immune-compatibility, teratoma formation) issues must be overcome before using them in clinical practice (Ben-David and Benvenisty, 2011). The recently discovered human iPSCs avoid the ethical issues of ESCs by generating autologous patient-specific cells. Unfortunately, iPSCs share the tumorigenicity characteristics found in ESCs (Ben-David and Benvenisty, 2011). Indeed, when iPSCs are delivered through intracerebroventricular (ICV) injection into an ischemic rat brain, large teratomas form, and there is little behavioral improvement compared with PBS-implanted controls rats (Kawai et al., 2010). However, new neuroblasts and mature neurons have been observed in the ischemic area, revealing a direct differentiation of iPSCs (Kawai et al., 2010). Following *in vitro* pre-differentiation of iPSCs into neuroepithelial-like stem cells, no tumors were found and improvements in function, a reduced infarct size and differentiated neuronal cells were reported (Jiang et al., 2011; Oki et al., 2012; Yuan et al., 2013; Eckert et al., 2015). These data indicate that iPSCs are another source of stem cells—beside adult NSCs—able to improve stroke recovery.

Among the different stem cell types that are candidates for grafting after stroke, MSCs represent the most promising candidates. Transplantation experiments with MSCs derived from different species (rats, mice, rabbit, or human) and using different routes of administration have been performed (Chen et al., 2001a,b; Horita et al., 2006; Cui et al., 2007; Gutiérrez-Fernández et al., 2011; Braun et al., 2012; Ruan et al., 2013). Stereotaxic (ST) transplantation of adult MSCs directly into the adult brain significantly reduces the functional deficit associated with stroke (Li et al., 2000; Kang et al., 2003; Horita et al., 2006). Moreover, significant reductions in infarct volume, as well as improvements in functional outcomes have also been observed following intravenous (IV) delivery of MSCs in a rodent model of stroke (Kurozumi et al., 2005; Honma et al., 2006; Koh et al., 2008). The mechanisms underlying the beneficial effects of MSCs are multifactorial. MSCs secrete numerous growth factors and cytokines that are neurotrophic, enhance revascularization and exhibit immunomodulatory properties as well as enhancing host neurotrophic factor expression, host neurogenesis and cell replacement (Kurozumi et al., 2005; Andrews et al., 2008; Bao et al., 2011, 2013). However, the contribution of grafted cells to the replacement of lost neurons is still unclear (Chen et al., 2001a,b). The efficacy of MSC grafts is largely time-dependent. Indeed, earlier MSC transplantations are associated with better functional recovery after stroke (Lee et al., 2015). This may be linked to the decreased inflammatory processes and the secretion of trophic factors by MSCs that reduce cellular apoptosis in the early period of stroke (Wang et al., 2014).

Altogether, stem cell graft experiments have highlighted promising potential strategies regarding stroke recovery, by either directly replacing lost neurons or more importantly, by helping endogenous proliferation and modulating inflammation.

### Endogenous stem cells

Even without any treatment, some degree of spontaneous recovery occurs after brain injury. Recent findings have shed light on the possibility that therapeutic outcomes after stroke may originate from endogenous NSCs residing in the adult brain, such as in the SVZ and the SGZ. Adult neurogenesis occurring in these areas could participate in the replacement of neurons lost following ischemia. Importantly, stroke induces cell proliferation in these specific areas, but also in other parts of the brain. These new neurogenic zones could potentially represent a “reservoir” of endogenous cells able to increase their proliferation after ischemic injury in order to repopulate damaged parenchyma. However, not much is known about the mechanisms underlying stroke-induced neurogenesis, in terms of cellular origin, molecular regulation or functional integration.

#### Localization

A basal rate of proliferation is present in the SVZ, SGZ and hypothalamus of the healthy adult mammalian brain. Therefore, the first experiments performed regarding stroke-induced neurogenesis have tried to determine from which part of the brain stroke-activated endogenous stem cells may come from.

##### Classical neurogenic niches: SGZ&SVZ

If endogenous stroke-induced neurogenesis occurs, the two most likely areas where it may happen are the two classical neurogenic niches SGZ and SVZ. In the hippocampus, NSCs—also named type-1 radial glia-like cells—are found in the SGZ, at the interface of the hilus and the granular cell layer (GCL). These cells divide slowly and give rise to type-2 cells or transit-amplifying progenitors, which divide actively to generate type-3 cells or neuroblasts. Neuroblasts exit the cell cycle and migrate a short distance into the GCL where they differentiate into immature postmitotic neurons. Around 50% of the immature neurons produced will die, and only a few newborn granule cells will be stably integrated into the synaptic network of the DG (Genin et al., 2014). This adult hippocampal neurogenesis is likely implicated in cognitive processes such as learning, memory, and cognition. Experimental models of cerebral ischemia are categorized as global or focal. In models of global ischemia, in which CBF is reduced throughout most of the brain, a 10-fold increase in SGZ progenitor proliferation has been demonstrated in many species, such as gerbils, rats, mice, monkeys as well as humans (Imai et al., 2007; Wiltrout et al., 2007). Focal ischemia models, which are more frequently used, consist of a temporary occlusion of the middle cerebral artery (MCAO) that produces infarcts in the ipsilateral area of the cerebral cortex and striatum (Ginsberg and Busto, 1989). In these models, studies have also reported a significantly enhanced proliferation of NSCs and progenitors in the SGZ (Yagita et al., 2001; Sharp et al., 2002; Wiltrout et al., 2007). Generally, the increased proliferation starts bilaterally at 3–4 days post-ischemia (Lichtenwalner and Parent, 2006), peaks at 7–10 days and returns to control levels by 3–5 weeks (Arvidsson et al., 2002; Takasawa et al., 2002; Dempsey et al., 2003; Zhu et al., 2003). One week after the ischemic episode, a 2–3-fold increase of cell production is observed in the DG as compared to the basal level seen in control animals. Over 60% of these cells express Calbindin—a calcium-binding protein normally expressed in mature granule neurons—5 weeks after the ischemia (Kee et al., 2001). Altogether, these data indicate that SGZ is likely to be one source of the endogenous stem cells responding to stroke.

The SVZ lies adjacent to the lateral ventricles along the lateral wall. It is composed of different types of NSCs classified by their self-renewal and differentiation capacities. Radial glial NSCs—the so-called type B cells—extend an apical ending that is exposed to the ventricle and possess a long basal process ending on blood vessels (Mirzadeh et al., 2010). These cells are surrounded by multiple ependymal cells (type E cells) forming pinwheel structures on the ventricle surface (Mirzadeh et al., 2008). Type B cells divide slowly to generate transit-amplifying type C cells, which proliferate actively and further differentiate into neuroblasts also named type A cells. Finally, these cells form chains and migrate over long distances toward the olfactory bulb (OB), via the rostral migratory stream (RMS). In the OB, neuroblasts migrate radially and differentiate into granule cells or periglomerular interneurons (Carleton et al., 2003).

There are numerous studies demonstrating that stroke stimulates SVZ NSCs proliferation and neurogenesis (Arvidsson et al., 2002; Kokaia and Lindvall, 2003; Thored et al., 2006). Some have demonstrated a post-MCAO activation of SVZ NSCs in rats (Jin et al., 2001; Zhang et al., 2001, 2004, 2007; Li et al., 2002; Parent et al., 2002), mice (Carlén et al., 2009; Zhang et al., 2014) and even monkeys (Tonchev et al., 2005) with an increase in proliferation markers that peaks at day 14 post-stroke. Beside this increased proliferation, it has been shown that these SVZ NSCs are migrating, differentiating and integrating into the lesion site tissue in rodent MCAO models (Zhang et al., 2001; Parent et al., 2002; Yamashita et al., 2006; Magnusson et al., 2014). Indeed, many BrdU+/NeuN+ cells were found in the post-ischemic striatum brain (Arvidsson et al., 2002) and a fluorescent tracing of SVZ proliferating cells showed that these cells directly migrate from the SVZ to the striatum in the post-ischemic rat brain (Jin et al., 2003). Ischemia may induce these newly generated neural precursors of the SVZ to revoke their normal migratory pattern, and instead to migrate toward the injured areas of the brain and aid in spontaneous recovery (Thored et al., 2006). Moreover, besides stroke-induced NSCs proliferation, non-proliferative type E cells have been shown to also be able to generate neuroblasts and astrocytes following ischemic injury (Carlén et al., 2009).

##### Novel neurogenic niches

Recent evidence suggests that, in addition to the canonical SGZ and SVZ, other stem cell niches are present in the adult brain. Indeed, the presence of adult NSCs has been proven in rodent hypothalamus (Kokoeva et al., 2005; Xu et al., 2005; Migaud et al., 2010; Pérez-Martín et al., 2010; Pierce and Xu, 2010) and also in the striatum of rats (Dayer et al., 2005), mice (Shapiro et al., 2009), rabbits (Luzzati et al., 2006), and monkeys (Bédard et al., 2006). Evidence for adult neurogenesis has also been shown in certain neocortical areas of the adult rat (Dayer et al., 2005), mouse (Shapiro et al., 2009) and monkey brain (Bernier et al., 2002). Finally, amygdala neurogenesis has been demonstrated in mice (Shapiro et al., 2009), rabbits (Luzzati et al., 2006), and monkeys (Bernier et al., 2002) as well as brainstem neurogenesis in rat (Bauer et al., 2005).

**Figure 1:**
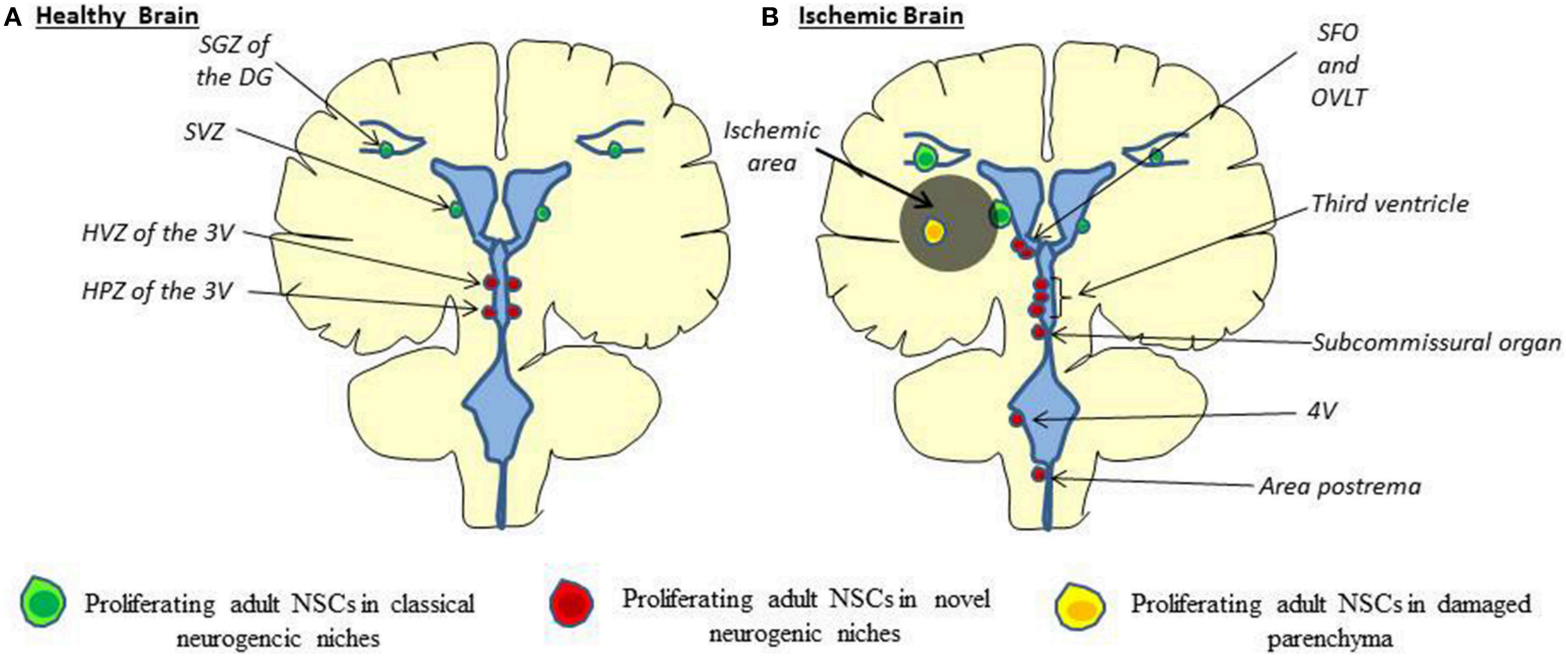
**Cellular origin of stroke-induced neurogenesis. (A)** In the healthy brain, adult neural stem cells are found to proliferate in the SGZ of the DG, in the SVZ along the lateral ventricle (green) and the HVZ and HPZ along the third ventricle (red). **(B)** In the ischemic brain, proliferating adult neural stem cells are found along the third ventricle, the fourth ventricle (4V) and the CVOs (SFO, OVLT, Subcommissural organ, area postrema) (Red) as well as directly in the ischemic parenchyma (orange), besides classical neurogenic niches (Lin and Iacovitti, 2015). HVZ, hypothalamic ventricular zone; HPZ, hypothalamic proliferating zone; CVOs, Circumventricular organs; SFO, subfornical organ; OVLT, organum vasculosum of the lamina terminalis.

*Hypothalamus.* The most described new stem cell niche is the hypothalamus. Proliferating cells found in the hypothalamus can be divided in two groups. The first one is located in the lateral wall of the third ventricle at the level of paraventricular, ventromedial and arcuate nuclei (Hypothalamic Ventricular Zone, HVZ, Figure 1). The second group, formed by tanycytes—a specialized ependymal cell type—is located at the bottom of the third ventricle in median eminence region and called the hypothalamic proliferating zone (HPZ). Physiologically, hypothalamic neurogenesis seems to play a role in energy balance; however, the rate of proliferation is much less than in the two canonical neurogenic niches (Rojczyk-Golebiewska et al., 2014). Nevertheless, tanycytes that form the rat and human HVZ express classical markers of neural precursor cells such as nestin (Wei et al., 2002) and doublecortin-like protein, a microtubule-associated protein highly homologous to doublecortin (DCX), a marker of neuroblasts and immature neurons, in the adult mouse brain (Saaltink et al., 2012). Importantly, proliferation of NSCs is enhanced on the ischemia ipsilateral side along the third ventricle (Lin et al., 2015).

*Ventricular system: windows of the brain.* Besides the SVZ and the median eminence, recent studies suggest that NSCs are also present at other circumventricular brain regions, particularly the sensory “circumventricular organs” (CVOs; Bennett et al., 2009; Furube et al., 2015). The sensory CVOs are the organum vasculosum of the lamina terminalis (OVLT), the subfornical organ (SFO), the pineal gland (PG), the subcommissural organ (SCO), and the area postrema (AP). The sensory CVOs, located outside the blood-brain barrier, are involved in the maintenance of a wide variety of sensory homeostatic and inflammatory pathways in the brain (Joly et al., 2007; Price et al., 2008). *In vivo* studies have revealed that CVOs cells proliferate and undergo constitutive neurogenesis and gliogenesis (Bennett et al., 2009). In addition to CVOs, proliferating cells are also found along the fourth ventricle (4V) (Lin et al., 2015). Consistent with these findings, neurospheres have been generated *in vitro* from isolated CVO and 4V cells (Bauer et al., 2005; Charrier et al., 2006; Itokazu et al., 2006).

Adult CVOs stem cells residing in a subventricular location incorporate BrdU and express the proliferation marker Ki67, as well as stem cells markers such as nestin and Sox2, as is the case in the SVZ (Bennett et al., 2009). Moreover, these cells are able to generate new neurons *in vivo*. Indeed, some proliferating cells found in CVOs were also positive for TUC-4 (TOAD (Turned On After Division)/Ulip/CRMP-4), a very early neuronal marker, and the mature neuronal marker NeuN (109). More importantly, in response to stroke in rats, an increased proliferation of NSCs in the CVOs as well as along the 4V has been observed. Similarly, proliferating cells are also observed in CVOs after stroke in humans (Sanin et al., 2013). CVO and 4V cells—like classical NSCs—are able to differentiate into oligodendrocytes, astrocytes, and neurons following stroke (Lin and Iacovitti, 2015) even if a shift toward neurogenesis was observed (Bennett et al., 2009). Interestingly, some newborn cells coming from novel neurogenic niches have been observed in a chain formation potentially migrating away from these novel niches to the infarct core (Lin et al., 2015). Taken together, the CVO reservoir of stem cells may serve as a further source of NSCs in humans for recovery after stroke.

##### Non-neurogenic niches

The increased proliferation and migration of endogenous stem cells in the neurogenic niches contributes to stroke-induced neurogenesis, and provides a clear link between stroke and neurogenesis. However, not only neurogenic niches must be taken into account regarding stroke-induced neurogenesis. Accumulating evidence suggests that ischemic injury induces the generation of new neurons from activated NSCs directly in the cerebral cortex (Magavi et al., 2000; Jiang et al., 2001; Jin et al., 2006; Yang et al., 2007). These ischemia-induced NSCs generate nestin-positive neurospheres *in vitro*. Following stroke, these nestin-positive cells develop in the ischemic subpial region, in proximity to blood vessels, and spread into the cortex through cortical layer 1 (Ohira et al., 2010; Nakagomi et al., 2011). These progenitor cells may originate from microvascular pericytes, as they express pericyte markers such as platelet-derived growth factor receptor β (PDGFRβ) and NG2 (Nakagomi et al., 2015). Indeed, new evidence has highlighted the multipotential differentiation capacity of vascular pericytes (PCs). PCs extracted from ischemic regions from the mouse or human brain and cultured under oxygen/glucose deprivation have developed stem cell-like features, presumably through reprogramming (Nakagomi et al., 2015). Moreover, cells from the adult human cerebral cortex that express PC markers, such as PDGFRβ and NG2, have been reprogrammed into neuronal cells by retrovirus-mediated co-expression of Sox2 and Mash1 (Karow et al., 2012). These induced-neuronal cells acquire the ability to fire repetitive action potentials and serve as synaptic targets for pre-existing neurons, indicating their capacity to integrate into neural networks. Taken together, these data show that PCs constitute a promising source of NSCs that can be activated following stroke and aid the replacement of dead neurons.

Besides PCs, recent studies have shown that under certain conditions, adult striatal parenchymal NG2-positive glial cells—i.e., oligodendrocytes precursors cells (OPCs)—are also able to generate neuronal progeny. Genetic fate mapping of individual DCX-positive cells present in the damaged parenchyma has revealed that some are of NG2^+^-glial origin. Furthermore, overexpression of Sox2 is sufficient to induce the conversion of genetically fate-mapped NG2^+^ glia into DCX+ cells in the adult mouse cerebral cortex following stab wound injury *in vivo* (Heinrich et al., 2014). Finally, it has also been demonstrated *in situ* in the brain that striatal NG2^+^ cells, transfected with Ascl1, Lmx1a and Nurr1, can be reprogammed into functional neurons that integrate into the local circuitry (Torper et al., 2015).

Unexpectedly, reactive astrocytes generated following stroke also appear to be an important source of endogenous cells that aid recovery. Although parenchymal astrocytes do not divide in the healthy brain and do not form neurospheres *in vitro*, the behavior of these cells drastically changes following brain injury. Following stroke, two types of reactive astrocytes have been identified: 1/reactive elongated astrocytes, which incorporate BrdU and express progenitor markers such as SOX2 and brain lipid binding protein (BLBP), and 2/stellate astrocytes that derive from resident cortical astrocytes (Wanner et al., 2013). A proportion of these reactive astrocytes has the potential to self-renew and is multipotent *in vitro* (Lang et al., 2004). Moreover, in addition to the conversion of local parenchymal astrocytes, it has been shown that a subpopulation of reactive the astrocytes generated following stroke is SVZ-derived and contributes to the astrocyte scar (Benner et al., 2013).

The ability of reactive astrocytes to generate neurons also depends on their location. Genetic and viral lineage tracing studies have reported the generation of neurons from reactive striatal astrocytes following MCAO (Magnusson et al., 2014; Duan et al., 2015). Conversely, there is a lack of evidence for a direct *in vivo* neurogenic potential of these reactive astrocytes in the cortex, probably due to anti-neurogenic influence. Nevertheless, a latent neurogenic program is present in cortical astrocytes. Indeed, a recent study has shown that SVZ-derived NSCs are able to give rise to reactive astrocytes at the stroke site and can be converted to neurons *in vivo* following overexpression of Ascl1 (Faiz et al., 2015).

The capacity for parenchymal cells—as pericytes, NG2-positive OPCs and reactive astrocytes—to give rise to neurons following stroke opens new perspectives for their use as a repair tool for brain regeneration.

#### Molecular cues

**Figure 2:**
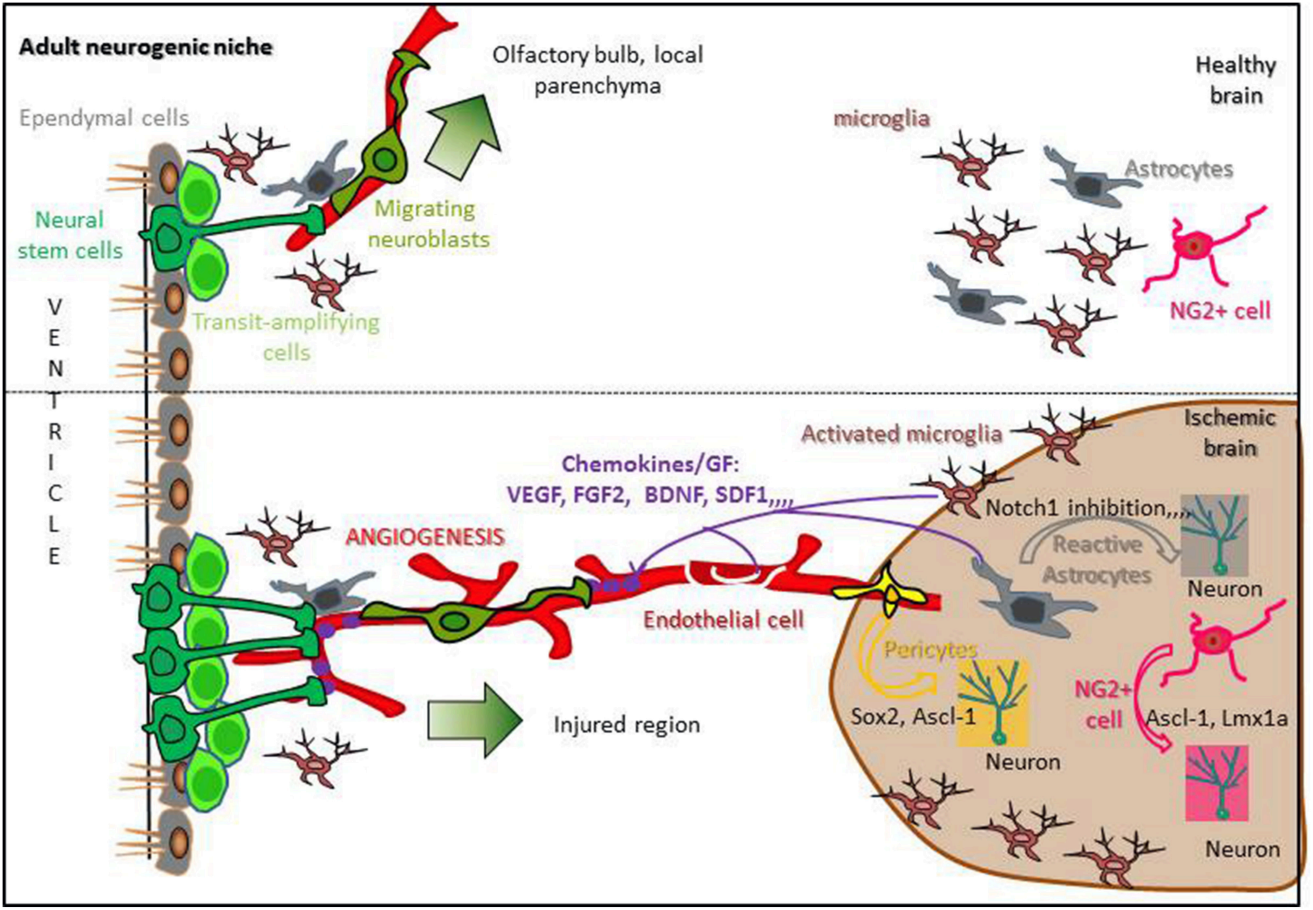
**Regulation of stroke-induced neurogenesis in a standard neurogenic niche**. In the healthy brain, adult neural stem cells (Dark green) present in neurogenic niches contact blood vessels, proliferate and give rise to neuroblasts migrating from the SVZ to the olfactory bulb or from an other neurogenic niche to the local parenchyma (upper panel). Following stroke, reactive astrocytes, activated microglia, and endothelial cells release chemokines and growth factors able to reach neurogenic niches, increasing NSCs proliferation and attracting migrating neuroblasts to the ischemic area. Moreover, reactive astrocytes, NG2+ cells and pericytes are able to dedifferentiate into neurons inside the damaged parenchyma (lower panel; Hermann et al., 2014; Sawada et al., 2014).

Spontaneous recovery following stroke may come 1/from the capacity of endogenous stem cells to proliferate into neurogenic niches, migrate toward the damaged parenchyma, and differentiate into neurons or 2/from parenchymal cells that proliferate, dedifferentiate into neuronal cells and survive to replace dead neurons. Understanding the molecular mechanisms regulating these processes is necessary to improve brain recovery. If neurogenic niches are a potent reservoir of NSCs that are activated following stroke, how these cells are instructed to proliferate and migrate long distances, far from the ischemic parenchyma, remains an open question (Figure 2).

##### Neurogenic niches

*Proliferation.* The initial response of NSCs following stroke is to increase proliferation, a process that is regulated by different environmental and molecular cues. Indeed, following stroke, the neurovascular unit combines with astrocytes, microglia and others mediators to create a favorable environment for NSCs proliferation in the neurogenic niches.

- Neurovascular regulation

Stroke induces dramatic changes in the vasculature (Sawada et al., 2014) and increases angiogenesis, defined as the formation of new capillaries from pre-existing vessels, suggesting a link between stroke-induced neurogenesis and blood vessels (Ruan et al., 2015). Following MCAO, reactive angiogenesis remodels the disrupted blood vessel network in the injured striatum, during a period of several days to 2 weeks after ischemia (Thored et al., 2007). Angiogenesis is found in the penumbra of the brain infarcted region in both animal models of stroke and in patients, and a positive correlation exists between survival and the density of new microvessels (Krupinski et al., 1994; Hayashi et al., 2003; Ruan et al., 2015).

This neo-angiogenesis leads to vasculature and blood flow changes. As a consequence, the levels of metabolic and gas molecules are either increased (ATP) (Suyama et al., 2012), or decreased (NO and O_2_) (Matarredona et al., 2005; Panchision, 2009) around the blood vessels, and this regulates the proliferation of adjacent NSCs (Lacar et al., 2012). Moreover, fluid dynamics can also regulate the active transport of blood-derived factors, such as insulin growth factor- 1 (IGF-1), to control adult SVZ neurogenesis (Nishijima et al., 2010). Consistent with the important role of angiogenesis in the regulation of stroke-induced NSC proliferation, blocking post-ischemic angiogenesis results in a 10-fold reduction in neuroblasts in the peri-infarct cortex 7 days after stroke (Ohab et al., 2006). Finally, angiogenesis leads to changes in the expression of growth factors, chemokines and metalloproteinases that can induce NSCs proliferation. Indeed, activated endothelial cells in the ischemic area secrete VEGF, which promotes NSCs proliferation and neuronal differentiation (Jin et al., 2002; Shen et al., 2004). In response to the ischemic injury, endothelial cells also secrete BDNF, erythropoietin, IGF-1, and FGF-2, which have been shown to significantly increase progenitor cell proliferation in both the SVZ and SGZ (Wiltrout et al., 2007). Interestingly, in the SVZ, NSCs are able to promote angiogenesis by secreting several angiogenic factors such as VEGFR2, Angiopoietin-1 and FGF-2 (Liu et al., 2007). This provides another strong piece of evidence that links stroke-induced neurogenesis and angiogenesis.

Besides angiogenesis, an important feature of neurogenic niches regarding stroke-induced proliferation is their location. Indeed, the majority of endogenous NSCs are directly or indirectly in contact with the CSF and other critical factors. In the SVZ, type B cells have direct access to the lumen of the ventricles, and CVO stem cells are directly located along the 3V and 4V. In contrast, the SGZ has no direct contact with the ventricle, but the hippocampal sulcus (the groove between the DG and CA1 field) could serve as a potential point of access to CSF factors. Another common feature of neurogenic niches is the rich network of blood vessels that surround and communicate with stem cells. In the SVZ, type B cells extend basal processes that terminate directly on endothelial cells (Figure 2). Importantly, these vessels lack the astrocyte endfeet and endothelial tight junctions that are found elsewhere in the brain, resulting in a highly permeable blood brain barrier (BBB) at the niche site (Shen et al., 2008; Tavazoie et al., 2008). CVOs are often called the “windows of the brain.” As a result, NSCs have access to both local endothelial cell-derived factors and systemic factors present in the blood stream, including circulating cytokines, chemokines and growth factors that all participate in adult neurogenesis (Goldberg and Hirschi, 2009; Lin and Iacovitti, 2015). Indeed, in physiological conditions, capillaries in the SVZ are known to be permeable to diffusible molecules released from endothelial cells, such as VEGF and pigment epithelium-derived factor (PEDF), which regulate NSCs proliferation (Jin et al., 2002; Andreu-Agulló et al., 2009). Moreover, adult NSCs express integrins (alpha6 beta1 and alphaVB8), which serve as receptors for laminin and TGFβ present within the vascular basement membranes. Activation of these pathways in endothelial cells leads to the production of growth factors that are necessary for NSC survival and function (Shen et al., 2008; Mobley et al., 2009). Moreover, growth factors, or reagents that stimulate their expression, increase stroke-induced SVZ neurogenesis, and conversely stroke-induced neurogenesis is impaired when growth factors concentrations are decreased (Chen et al., 2005; Tsai et al., 2006; Yan et al., 2006).

- Other regulators

In neurogenic niches, microglia are abundant and in close contact with adult NSCs. Microglia activated after ischemic brain injury are also able to produce chemokines and cytokines that aid NSCs proliferation (Gonzalez-Perez et al., 2010; Yenari et al., 2010). Notably, activated microglia are able to secrete IGF-1 to promote NSCs proliferation in both of the classical neurogenic niches (Hwang et al., 2004; Thored et al., 2009). Consistent with this, inhibition of microglial activation after cerebral ischemia decreases stroke-induced proliferation in the SVZ (Kim et al., 2009).

Finally, other potential mediators of stroke-induced proliferation neurogenesis—mainly in the classical neurogenic niches—have been described. These include Notch signaling (Wang et al., 2009), retinoic acid (Plane et al., 2008), bone morphogenetic protein (Chou et al., 2006), tumor necrosis factor-alpha (Iosif et al., 2008), and sonic hedgehog (Sims et al., 2009). Besides chemokines and soluble factors, other regulators of SVZ and DG stem cell proliferation have been described, such as microRNA (Liu et al., 2013), exercise (Luo et al., 2007), and electroacupuncture (Kim et al., 2014).

*Migration.* Following NSCs proliferation, the next step following stroke is the production of new neurons in the injured region. Indeed stroke can induce long-distance cell migrations of newly born immature neurons to the peri-infarct cortical area (Tsai et al., 2006; Yamashita et al., 2006). Studies have shown that following BrdU or GFP lentiviral injections into the SVZ at the time of stroke, labeled cells are detected in peri-infarct cortex at 7 and 14 days post stroke, whereas injections of BrdU directly into the cortex results in only a few BrdU+/DCX+ cells. Several critical regulators of newborn cells following stroke have been identified over the years.

- Vascular regulation

Similar to the regulation of stroke-induced NSCs proliferation, the vascular environment is also a key feature regarding the migration of newborn cells following stroke.

• *Scaffold cues*

During embryonic development and early postnatal stages, coordinated neurogenesis and angiogenesis are important (Sawada et al., 2014). Indeed, at the neonatal stage, SVZ-derived newborn cells migrate along blood vessels not only to the OB but also to the cerebral cortex (Le Magueresse et al., 2012). This blood vessel-guided cell migration toward the cerebral cortex gradually decreases during postnatal development, probably due to a decrease in blood vessel density in the corpus callosum (Le Magueresse et al., 2012). Following stroke, this developmental cell migration toward the cortex is reactivated, and chains of neuroblats migrate in the direction of the damaged parenchyma, along the blood vessels away from both the classical and novel niches (Pencea et al., 2001; Sawada et al., 2014; Lin et al., 2015).

Post-stroke neuroblast migration toward the injured region shares characteristic features with physiological neuroblast migration within the RMS. In both cases, neuroblasts form chain-like cell aggregates (Arvidsson et al., 2002; Parent et al., 2002; Yamashita et al., 2006) and migrate along blood vessels (Zhang and Chopp, 2009; Kojima et al., 2010; Saha et al., 2013). After stroke, during the blood vessel-guided migration, newborn neuroblasts are frequently associated with thin astrocytic processes (Jin et al., 2003; Thored et al., 2006; Yamashita et al., 2006; Kojima et al., 2010), which directly contact vascular endothelial cells (Le Magueresse et al., 2012), forming a neurovascular niche. Double labeling for BrdU and PECAM-1 confirms that blood vessels in the peri-infarct cortex contain newly born vascular endothelial cells (Lacar et al., 2012). Moreover, inhibiting angiogenesis decreases the number of new neurons in injured regions (Taguchi et al., 2004; Ohab et al., 2006; Cayre et al., 2013), suggesting that newly generated blood vessels play a role in neuronal regeneration. Since new neurons migrate along both pre-existing and newly generated blood vessels after MCAO, both old and new blood vessels appear to act as a migratory scaffold for new neuroblasts moving toward injured regions (Kojima et al., 2010; Grade et al., 2013). Thus, remodeling the blood vessel network in injured regions and regulating the direction of new neuron migration could improve the efficiency of blood vessel-guided neuronal migration and neuronal regeneration.

• *Guidance cues*

Besides their role as scaffolds, endothelial cells are also able to secrete several factors that regulate the migration of neuroblasts, both in normal and pathological conditions. During adult neurogenesis, stromal-derived factor-1α (SDF-1α), a CXC chemokine, is secreted by vascular endothelial cells and enhances the motility of neuroblasts, which express its receptor CXR4, toward the RMS (Kokovay et al., 2010). Similarly, following stroke, endothelial cells and reactive astrocytes upregulate SDF-1 (Ohab et al., 2006; Thored et al., 2006), which guides neuroblast migration toward the peri-infarct region (Robin et al., 2006). Administration of SDF-1 improves behavioral recovery during the period in which immature neurons migrate (Ohab et al., 2006), whereas blockade of CXRC4 with the specific antagonist AMD3100 alters the migration of new neurons *in vitro* and *in vivo* (Robin et al., 2006; Thored et al., 2006; Kojima et al., 2010).

- Astrocytes/microglia

Activated astrocytic and microglial populations in the core and the penumbra produce several factors, including cytokines and chemokines, which may act as putative chemoattractants for proliferating progenitors. For example, the expression of monocyte chemoattractant protein-1 (MCP-1), a CC chemokine, is induced in activated microglia and astrocytes after MCAO, while migrating neuroblasts express the MCP-1 receptor CCR2 (Cayre et al., 2013). Furthermore, MCP-1 and CCR2 KO mice display a significant decrease in the number of migrating neuroblasts from the ipsilateral SVZ to the ischemic striatum (Yan et al., 2007).

- Other regulators

Matrix metalloproteinases (MMPs) are expressed by endothelial cells following stroke, and digest the extracellular matrix to enable migrating newborn cells to penetrate the damaged parenchyma. MMPs have been implicated in guiding neuroblast migration from the neurogenic region to the ischemic boundary (Grade et al., 2013). MMP9 is upregulated in the infarcted cortex and co-localizes with DCX+ and BrdU+ cells migrating from SVZ. Moreover, blocking the activation of MMPs severely diminishes striatal migration (Lee et al., 2006; Barkho et al., 2008).

##### Non-neurogenic niches

As discussed above, neurogenesis also occurs in the damaged parenchyma. However, it seems that an anti-neurogenic environment prevents reactive astrocytes from becoming neurons. A more complete understanding of the molecular mechanisms that determine gliogenesis vs. neurogenesis is important to increase the number of newborn neurons in the injured area. Notably, the oligodendrocyte transcription factor OLIG2 and the neurogenic paired-box protein PAX6 are both potent regulators of stroke-induced neurogenesis. Stereotaxic retroviral injection of either a dominant negative form of Olig2, or a PAX6 overexpression construct, in the lateral striatum of MCAO rat brains results in a significant increase in the number of DCX-expressing immature neurons (Kronenberg et al., 2010; Robel et al., 2011). In addition, inhibition of Notch signaling—a strong anti-neurogenic pathway—triggers astrocytes in the striatum and the medial cortex to enter a neurogenic program, even in physiological conditions (Magnusson et al., 2014). Moreover, TNF-α or noggin—a bone morphogenetic protein (BMP) inhibitor—can modulate the latent neurogenic capacity of parenchymal astrocytes (Michelucci et al., 2015). Therefore, modulating the fate of endogenous parenchymal astrocytes represents an interesting target to induce neurogenesis after stroke.

#### Functional impact

Several recent studies have shed light on the essential role of stroke-induced neurogenesis on functional recovery. Consistent with the neuroprotective role of NSCs, targeted depletion of both DCX- or Nestin-expressing cells in the SVZ has been linked to worsened stroke lesion size and motor impairment (Wang et al., 2012; Sun et al., 2013). On the contrary, manipulations aimed at increasing neurogenesis have been shown to improve functional outcomes (Leker et al., 2007). In endothelin-induced stroked rats, a combined treatment of fluoxetine, simvastatin and ascorbic acid produces a significant increase in neurogenesis, which is coupled to a strong functional recovery (Corbett et al., 2015). In ischemic mice brains, post-stroke chronic metformin treatment has been shown to enhance angiogenesis, neurogenesis and improve functional recovery following MCAO (Jin et al., 2014). These studies establish a clear link between stroke-induced neurogenesis and functional recovery. However, it is not yet understood why spontaneous endogenous neurogenesis does not lead to a complete recovery. Many milestones have to be reached in order to obtain functional recovery following stroke. Appropriate differentiation, long-term production and survival of stroke-induced newborn neurons, as well as the inflammatory response, are all potential restrictive characteristics regarding stroke recovery.

##### Differentiation and integration of newborn neurons

In order to contribute to functional recovery, nascent neurons, coming from non-neurogenic regions or neurogenic niches, must mature both morphologically and functionally. Early work showed that newly generated cells in the damaged striatum express markers of medium-size spiny neurons like DARPP-32, representing 95% of neurons within the striatum (Arvidsson et al., 2002; Parent et al., 2002). More recently, newborn cells, following focal cerebral ischemia, have also been shown to express appropriate neurotransmitter synthesizing enzymes such as glutamic acid decarboxylase (GAD67) and choline acetyl-transferase (ChAT). Moreover, these neurons exhibit electrophysiological activity and functional synapses (Hou et al., 2008). These data suggest that stroke-induced newborn GABAergic and cholinergic neurons can integrate into the striatal neural networks. However, if about 90–95% of striatal neurons are GABAergic medium-size spiny projection neurons, 5–10% of the remaining neurons are local interneurons that are classically divided into parvalbumin+ (PV+), calretinin+ (CR+), somatostatin+ (SOM+), and choline acetyltransferase+ (ChAT+) neurons (Marin et al., 2000). Immunostainings performed against these different markers, coupled with BrdU labeling, have shown that SVZ neuroblasts can produce CR-expressing newborn cells in the damaged striatum (Liu et al., 2009). Finally, another study has shown that newly born immature neurons differentiate into mature PV-expressing neurons, replacing more than 20% of PV+ interneurons lost after ischemia (Teramoto et al., 2003). Taken together, these data indicate that proliferating neuroblasts that migrate into the damaged striatum following stroke are able to differentiate into a variety of functional neuronal cells.

##### Long-term survival/production of newborn neurons

The long-term survival of newly-formed neurons following stroke is also crucial for a successful functional recovery. Although recent work indicates that new neurons persist for at least 3–4 months after stroke (Thored et al., 2006; Leker et al., 2007), survival of newly generated cells is inefficient (Turnley et al., 2014). Only 10% of the initial number of neuroblasts that migrate to the peri-infarct cortex express mature neuronal markers (Gu et al., 2000; Dempsey et al., 2003), while 6 weeks after ischemia, one third of DCX+/BrdU+ cells express mature neuronal markers (Thored et al., 2006). Moreover, many of these DCX+ cells co-expressing cPARP, a substrate of active caspases, which suggests there is widespread apoptosis within the DCX+ cell population (Thored et al., 2006). Taken together, these data indicate that only a small proportion of cells survive long enough to integrate into the damaged parenchyma. This may be linked to an inappropriate inflammatory response in the ischemic area that is deleterious for newborn neuron survival. In support of this, treatment with indomethacin, a non-steroidal anti-inflammatory agent, suppresses inflammation and microglial activation, and stimulates the accumulation of newborn neurons in the injured striatum following MCAO in adult rats (Hoehn et al., 2005). Moreover, up-regulation of TNFα expression following stroke has been shown to decrease SVZ progenitor proliferation, whereas blockade of TNF receptor-1 signaling has been demonstrated to increase stroke-induced SVZ cell proliferation and neuroblast formation (Iosif et al., 2008).

Initial studies suggested that increased SVZ neurogenesis is transient, as progressive recovery of certain behavioral deficits does not continue beyond 1 month. However, BrdU injections have shown a similar proportion of DCX+/BrdU+ at either at 2 or 8 weeks post-ischemia (Thored et al., 2006). Moreover, the migration of SVZ neuroblasts to the injured striatum may persist for up to 1 year after ischemia (Thored et al., 2007), suggesting that the SVZ may serve as a constant reservoir of new neurons offering a long-term window for therapeutic manipulations.

## Conclusion

Besides rtPA treatment, there is an urgent need to develop new treatments for stroke that are aimed at ultimately replacing dead neurons. While exogenous stem cell therapy presents interesting outcomes, increasing endogenous neurogenesis constitutes the most promising therapeutic strategy. Indeed, the presence of multiple pools of endogenous adult neuroblasts that are able to proliferate, migrate and differentiate, offers multiple possibilities for interventions. These strategies require that the as yet unknown molecular mechanisms that instruct stem cells to differentiate into specific neuronal cell types will also work in the brain of the affected individual. For maximum functional recovery, transplantation should probably be combined with a stimulation of neurogenesis from endogenous NSCs.

However, several critical questions have to be addressed before clinical trials can begin. Optimization of the timing and treatment is required, along with the identification of factors that give the most favorable survival and function of new cells, irrespective of their exogenous or endogenous origin. In addition, the heterogeneity among stroke patients constitutes an important challenge. The use of clinically relevant experimental animal models is essential, since numerous successful preclinical trials have failed to confirm their efficacy upon translation to humans. Moreover, inflammation is increasingly recognized as a key factor in stroke, but whether it is detrimental or beneficial depends on the severity and stage of the ischemia. It appears that an inflammatory response during the early stages of stroke potentiate ischemic injury, while late inflammation appears to be important for recovery and repair. Future work should focus on elucidating how the immune system moves from these damaging to protective/restorative responses. Consequently, a nuanced modulation of inflammation may lead to improved exogenous and endogenous potentiality regarding stroke recovery.

Elucidating the molecular mechanisms that regulate endogenous neurogenesis in stroke can be extended to other neurodegenerative diseases. Indeed, despite different triggering events, a common feature of neurodegenerative disease is neuronal cell death and immune responses.

## Conflict of interest statement

The authors declare that the research was conducted in the absence of any commercial or financial relationships that could be construed as a potential conflict of interest.
